# Semi-supervised learning with pseudo-labeling compares favorably with large language models for regulatory sequence prediction

**DOI:** 10.1093/bib/bbae560

**Published:** 2024-11-02

**Authors:** Han Phan, Céline Brouard, Raphaël Mourad

**Affiliations:** INRAE, MIAT, 31326 Castanet-Tolosan, France; INRAE, MIAT, 31326 Castanet-Tolosan, France; INRAE, MIAT, 31326 Castanet-Tolosan, France; University of Toulouse, UPS, 31062 Toulouse, France

**Keywords:** regulatory genomics, deep learning, semi-supervised learning

## Abstract

Predicting molecular processes using deep learning is a promising approach to provide biological insights for non-coding single nucleotide polymorphisms identified in genome-wide association studies. However, most deep learning methods rely on supervised learning, which requires DNA sequences associated with functional data, and whose amount is severely limited by the finite size of the human genome. Conversely, the amount of mammalian DNA sequences is growing exponentially due to ongoing large-scale sequencing projects, but in most cases without functional data. To alleviate the limitations of supervised learning, we propose a novel semi-supervised learning (SSL) based on pseudo-labeling, which allows to exploit unlabeled DNA sequences from numerous genomes during model pre-training. We further improved it incorporating principles from the Noisy Student algorithm to predict the confidence in pseudo-labeled data used for pre-training, which showed improvements for transcription factor with very few binding (very small training data). The approach is very flexible and can be used to train any neural architecture including state-of-the-art models, and shows in most cases strong predictive performance improvements compared to standard supervised learning. Moreover, small models trained by SSL showed similar or better performance than large language model DNABERT2.

## Introduction

Complex genetic diseases are pathologies that are caused by a set of mutations, lifestyle and environmental factors [1]. Those diseases are highly prevalent in the population and include some cancers, heart diseases, neurological disorders and autoimmune diseases [2–4]. Over the past 20 years, genome-wide association studies have comprehensively mapped thousands of single nucleotide polymorphisms (SNPs) associated with these diseases [1]. However, the majority of these SNPs were found outside of coding sequences, making it difficult to understand the underlying biological pathways [5].

Deep neural networks are increasingly used for regulatory element prediction from the DNA sequence. Convolutional neural networks (CNNs) were initially proposed, and achieved very good performances [6, 7]. Nevertheless, CNNs could not capture long-range interactions between distant DNA motifs. To tackle this issue, other models were proposed instead to model long-range interactions, including recurrent neural networks with LSTM for few hundred bases [8] and dilated convolutions [9] for hundreds of kilobases. Those models were classically trained with labeled data only (supervised learning), which are limited by the finite size of the human genome (3.3 Gb).

Self-supervised learning with large language models (transformers) were recently trained on DNA sequences without labeled data, as a pretraining step before a fine-tuning step [10–16]. While such approach showed great performances, the training of large models (>100M parameters) on a large quantity of data (several genomes) requires important computational ressources, i.e. a cluster of GPUs or TPUs. An alternative to self-supervised learning is semi-supervised learning (SSL), which uses during training a mix of labeled and unlabeled data [17, 18]. Unlike other natural languages, DNA sequences are texts that are phylogenetically conserved among species due to the evolution process. In particular, it is known that many regulatory sequences and their associated functions are strongly conserved between closely related species. Therefore, conservation of regulatory sequences between closely related genomes could be used to greatly increase the size of the data by pseudo-labeling of sequences.

Here, we propose a novel SSL method based on cross-species pseudo-labeling, which greatly augments the size of the available labeled data for learning [19–21]. The proposed method consists in remapping regulatory sequences from a labeled genome (e.g. human genome) to other closely related genomes (e.g. mammalian genomes). Pseudo-labeled data allows a neural network to be pre-trained on data several orders of magnitude larger than the original labeled data. After pretraing with pseudo-labeled data, the model is then fine-tuned on the labeled data. Combined with an approach inspired from the Noisy Student algorithm [22], the proposed method was further improved. The proposed SSL was used to train multiple state-of-the-art models, including DeepBind, DeepSea, and DNABERT2, and showed sequence classification accuracy improvement in many cases. We also found an improvement in SNP effect prediction with SSL, especially for specific TFs.

## Materials and methods

### Human experimental data

We used publicly available ATF3, ETS1, REST, MAX, P300, RAD21, CTCF, H3K4me3, POL2, and ANDR ChIP-seq data and input data of human lymphoblastoid GM12878 from Gene Expression Omnibus (GEO) accession GSE31477, GSE170139, GSE104399, GSE95899 from ENCODE [23]. We used publicly available ATAC-seq data of lymphoblastoid GM12878 from GEO accession GSE170918 from ENCODE [23]. All the data were mapped on hg38 and peak calling was done using macs3 [24].

### Human data labeling

#### Classification for benchmarking with DeepBind and DNABERT2

We binned the human genome (e.g. human genome assembly hg38) into non-overlapping genomic intervals of 200 b, where a given bin was considered as either bound by a TF (if overlapping $> 50\%$ a peak), or not bound otherwise. For each 200 b bin, we extracted the corresponding DNA sequence (only containing the bin). This approach is very similar to one used in DeepSea [7], except that we didn’t extract the surrounding DNA sequence of 1 kb (containing the bin and the context).

#### Classification for benchmarking with DeepSea

We used the same approach as in DeepSea [7], described as follows. We binned the human genome (e.g. human genome assembly hg38) into non-overlapping genomic intervals of 200 b, where a given bin was considered as either bound by a TF (if overlapping $> 50\%$ a peak), or not bound otherwise. For each 200 b bin, we extracted the surrounding DNA sequence of 1 kb (containing the bin and the context).

### Cross-species data labeling

To search for homologous sequences, we used the following 22 mammalian genomes: Rhesus monkey (rheMac10), marmoset (calJac3), chimpanzee (panTro6), pygmy chimpanzee (panPan2), Sumatran orangutan (ponAbe3), gorilla (gorGor6), olive baboon (papAnu4), crab-eating macaque (macFas5), Bolivian squirrel monkey (saiBol1), northern white-cheeked gibbon (nomLeu3), gray mouse lemur (micMur2), small-eared galago (otoGar3), mouse (mm10), rat (rn7), ferret (musFur1), rabbit (oryCun2), pork (susScr11), cat (felCat9), dog (canFam3), horse (equCab3), cow (bosTau9), and opossum (monDom5).

For pseudo-labeling, we liftovered peaks (ChIP-seq, ATAC-seq) from the human genome (labeled genome) to unlabeled genomes as follows. The UCSC liftover program was used to liftover the peak coordinates from the labeled genome to the unlabeled genomes. After liftover, a peak could be split into a set of non-overlapping regions. If a peak mapped to different loci in another genome, but the different loci remained close to each other (separated by < 20 b), the different loci were merged into one loci (homologous peak). Then, the corresponding DNA sequence was extracted and considered as a homologous sequence. If the peak mapped to distant loci (separated by >= 20 b) or if the peak did not map to any loci, then no homologous peak was found and no homologous sequence was extracted.

### Models

#### Shallow CNN

We have designed a simple shallow CNN (similar to DeepBind [6]) which consisted in a convolutional layer (64 or 256 filters and kernel${\_ }$size=24, activation=‘relu’), a 1D global max pooling layer, a dropout layer (dropout${\_ }$rate=0.2), a dense layer (10 units), a dropout layer (dropout${\_ }$rate=0.2) and a last classification layer (1 unit, activation=‘sigmoid’).

#### Deep CNN

We have also designed a more sophisticated CNN (similar to Basenji [9]), which consisted in a convolutional layer (64 or 256 filters and kernel${\_ }$size=24, activation=‘relu’), followed by five successive dilated convolutional layers with residual connection (64 or 256 filters and kernel${\_ }$size=3, activation=‘relu’), a 1D global max pooling layer, a dropout layer (dropout${\_ }$rate=0.2), a dense layer (10 units), a dropout layer (dropout${\_ }$rate=0.2), and a last classification layer (1 unit, activation=‘sigmoid’).

#### DeepBind

We reimplemented the DeepBind model using Keras, keeping the original model architecture [6], a convolutional layer (16 filters and kernel${\_ }$size=24, activation=‘relu’), a 1D global max pooling layer, a dense layer (32 units), a dropout layer (dropout${\_ }$rate=0.5), and a last classification layer (1 unit, activation=‘sigmoid’).

#### DeepSea

We reimplemented the DeepSea model using Keras keeping the original model architecture [7]: a convolutional layer (320 filters and kernel${\_ }$size=8, activation=‘relu’), a 1D max pooling layer (pool${\_ }$size=4, strides=4), a dropout layer (dropout${\_ }$rate=0.2), a convolutional layer (480 filters and kernel${\_ }$size=8, activation=‘relu’), a 1D max pooling layer (pool${\_ }$size=4, strides=4), a dropout layer (dropout${\_ }$rate=0.2), a convolutional layer (960 filters and kernel${\_ }$size=8, activation=‘relu’), a dropout layer (dropout${\_ }$rate=0.5), a flatten layer, a dense layer (925 units), and a last classification layer (1 unit, activation=‘sigmoid’).

#### DNABERT2

We fine-tuned DNABERT2 [12] (downloaded from zhihan1996/DNABERT-2-117M huggingface repository) for classification with the following default parameters as provided with the ‘config.json’ file: alibi${\_ }$starting${\_ }$size=512, architectures=‘BertForMaskedLM’, attention${\_ }$probs${\_ }$dropout${\_ }$prob=0.0, gradient${\_ }$checkpointing=false, hidden${\_ }$act=‘gelu’, hidden${\_ }$dropout${\_ }$prob=0.1, hidden${\_ }$size=768, initializer${\_ }$range=0.02, intermediate${\_ }$size=3072, layer${\_ }$norm${\_ }$eps=1e-12, max${\_ }$position${\_ }$embeddings=512, model${\_ }$type=‘bert’, num${\_ }$attention${\_ }$heads=12, num${\_ }$hidden${\_ }$layers=12, position${\_ }$embedding${\_ }$type=‘absolute’, torch${\_ }$dtype=‘float32’, transformers${\_ }$version=4.28.0, type${\_ }$vocab${\_ }$size=2, use${\_ }$cache=true, vocab${\_ }$size=4096. Moreover, we used low-rank adaptation for fine-tuning [25].

### Prediction of SNP effect

We used the previous deep learning models to predict the effect of SNPs on functional data, such as protein binding or chromatin accessibility. To compute the effect of an SNP, we used the following approach for classification as in [6]. First, model predictions were computed both for the DNA sequence comprising the reference SNP allele ($p_{ref}$) and the same DNA sequence but with the alternative SNP allele ($p_{alt}$). Then, the SNP effect was computed as $(p_{alt}-p_{ref}) \cdot max(0,p_{alt},p_{ref})$, where $p_{alt}$ is the prediction from the alternative allele and $p_{ref}$ the prediction from the reference allele.

**Figure 1:**
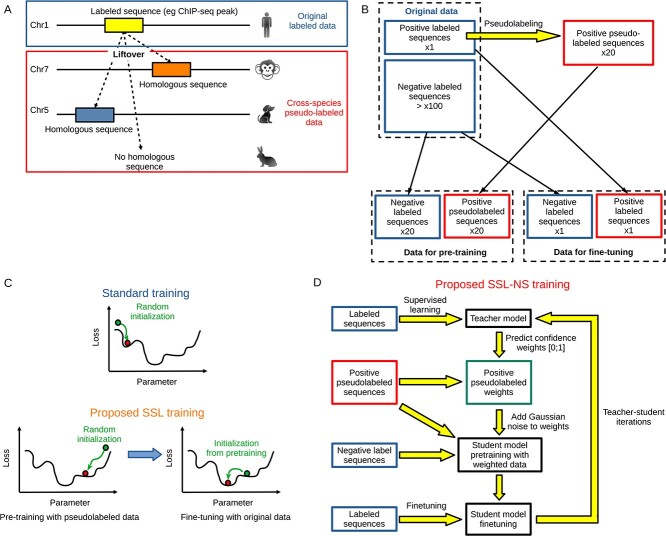
Data pseudo-labeling and associated SSL. (A) Data pseudo-labeling using cross-species labeling by homology. (B) Data used for pre-training and for fine-tuning. (C) SSL based on pre-training with pseudo-labeled data, followed by fine-tuning with the original labeled data (so called SSL). (D) SSL based on pre-training with pseudo-labeled data with a noisy-student-like approach (so called SSL-NS).

### Implementation and availability

The model was developed using Tensorflow and Keras. It is available at the Github repository: https://forgemia.inra.fr/raphael.mourad/deepssl

## Results and discussion

### Cross-species data labeling approach and novel training strategy

The proposed cross-species pseudo-labeling approach is illustrated in Fig. 1(A). Given a labeled sequence (e.g. ChIP-seq peak in yellow), cross-species data labeling consists in identifying the homologous sequences in related species (e.g. mammals) using liftover from the labeled genome (e.g. the human genome) to the unlabeled genomes (e.g. the chimpanzee, the dog, and the rabbit genomes). In Fig. 1(A), a homologous sequence was found in the chimpanzee (in orange) and in the dog (in blue), but no homologous sequence was found in the rabbit. Cross-species labeled data can then be used to ‘augment’ the original labeled data (e.g. the human genome), in sense that it can considerably increase the amount of labeled data that could be used for pre-training the model.

Using the cross-species pseudo-labeled data, we designed a novel SSL strategy to train more efficiently deep learning models. The strategy consists in two steps:

- first, pre-training of the model using cross-species pseudo-labeled data. For this purpose, the model is trained with balanced data comprising positive sequences corresponding to all sequences pseudo-labeled as overlapping a peak (i.e. non-human sequences that are homologous to human sequences overlapping a peak), and the same number of randomly drawn negative sequences corresponding to sequences labeled as non-overlapping a peak (i.e. human sequences) (Fig. 1B).
- second, fine-tuning of the model using the original labeled data (e.g. human labeled sequences). The model is trained with balanced data comprising positive sequences corresponding to sequences labeled as overlapping a peak (human sequences), and the same number of randomly drawn negative sequences corresponding to sequences labeled as non-overlapping a peak (i.e. human sequences) (Fig. 1B).

The rationale behind is that the pre-training step with pseudo-labeled data will provide a better parameter initialization for the fine-tuning with the original labeled data (Fig. 1C).

In addition, we have further improved our SSL using a training approach inspired from the Noisy Student algorithm [22], which we called SSL Noisy Student (SSL-NS) (Fig. 1D). First, a teacher model is trained on labeled sequences. The teacher model is used to compute confidence weights on positive pseudolabeled sequences (confidence weights are model outputs, ranging from 0 to 1). Then, a student model is pretrained using the set of weighted positive pseudolabeled sequences (weights are close to 1 for highly confident sequences and labels are set to 1) and the set of negative sequences (whose weights are all set to 1 and labels are set to 0). A Gaussian noise with mean=0 and SD=0.1 is added to the weights, which encourages the model’s decision making frontier to be smooth. The student model is then finetuned on labeled sequences. The student can next be used as a teacher model, and five teacher–student iterations were used.

### Improving state-of-the-art model performance with SSL

We then applied our novel SSL training scheme with three state-of-the-art models, namely: DeepBind (shallow CNN), DeepSea (deep CNN), and DNABERT2 (large language model). For each model, we compared prediction performances for sequence classification for different experimental data between standard supervised learning (i.e. without pretraining on pseudo-labeled data) and our novel SSL (i.e. with pretraining on pseudo-labeled data).

For a comprehensive benchmarking, we ran the comparison on diverse experiments:

- ChIP-seq of ATF3, a specific TF binding to 1677 loci with a median peak size of around 170 pb;
- ChIP-seq of ETS1, a specific TF binding to 4120 loci with a median peak size of around 224 pb;
- ChIP-seq of ANDR, a specific TF binding to 4638 loci with a median peak size of around 266 pb;
- ChIP-seq of REST, a specific TF binding to 6119 loci with a median peak size of around 276 pb;
- ChIP-seq of MAX, a specific TF binding to 13 605 loci with a median peak size of around 421 pb;
- ChIP-seq of P300, a specific TF binding to 14 223 loci with a median peak size of around 217 pb;
- ChIP-seq of RAD21, a wide-spread TF binding to 34 623 loci with a median peak size of around 265 pb;
- ChIP-seq of CTCF, a wide-spread TF binding to 72 779 loci with a median peak size of around 210 pb;
- ChIP-seq of H3K4me3, a histone modification that marks promoters and that is present at 25 641 loci with a median peak size of around 438 pb;
- ChIP-seq of POL2, a wide-spread multiprotein complex that transcribes DNA into pre-mRNA and that binds to 35 982 loci with a median peak size of around 250 pb;
- ATAC-seq, a non-specific assay that maps accessible chromatin and which was observed at 102 030 loci (or peaks) with a median peak size of around 230 pb.

The benchmarking was done in a classification setting (presence/absence of a peak in a bin). Models were trained on all chromosomes, except chromosomes 8 and 9 that were kept for testing. In Table 1, for each data, we show the number of positive sequences and the number of pseudo-labeled positive sequences obtained by pseudo-labeling on 22 mammalian genomes. For instance, for ATF3, a TF that binds to very few sequences (1306), the number of positive sequences obtained by pseudo-labeling was 23 555, which corresponds to an increase by $\times $18. Of note, pseudo-labeling with 22 mammalian genomes does not increase by $\times $22 the amount of labeled sequences, as there are many sequences that are not evolutionary conserved (Table S1 shows the number of positive sequences obtained by pseudo-labeling for each mammalian genome). However, such approach is very efficient, as, on average, $\frac{18}{22}=82\%$ of sequences are conserved and therefore could be pseudo-labeled.

**Table 1:** Number of positive sequences and pseudo-labeled positive sequences obtained by pseudo-labeling with 22 mammalian genomes for each data

Data	Number of positive sequences	Number of pseudo-labeled positive sequences	Increase
ATF3	1306	23 555	18x
ETS1	4211	77 661	18x
ANDR	4638	36 164	8x
REST	7201	130 615	18x
MAX	23 916	441 295	18x
P300	15 844	272 923	17x
RAD21	37 113	646 495	17x
CTCF	75 082	1 245 195	17x
H3K4me3	67 589	1 220 489	18x
POL2	57 226	998 107	17x
ATAC	70 600	1 186 950	17x

#### Implementation with DeepBind (CNN), DeepSea (CNN), and DNABERT2 (LLM)

**Figure 2:**
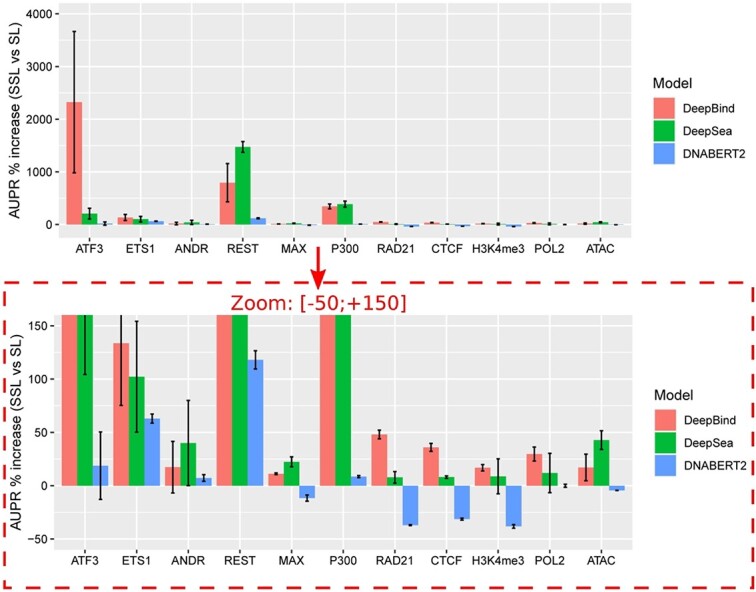
Percent of AUPR increase using SSL as compared to SL. Benchmarking was done for DeepBind, DeepSea, and DNABERT2. Each model was run three times and the average statistics were computed.

We first comprehensively compared classical supervised learning (SL) with our novel SSL training for DeepBind. In term of area under the receiver operating characteristic (AUROC) curve (Fig. S1), we observed an overall significant increase using SSL ($p$=$2 \times 10^{-9}$). There was a large increase with SSL compared to SL for ATF3 ($+11.3\%$), REST ($+7.4\%$), and P300 ($+24.4\%$). But for other experiments, gains were slight, around $1-2\%$. However, as the data were highly inbalanced with more negative sequences, the area under the precision recall (AUPR) curve was more appropriate. In term of AUPR (Fig. 2), improvements were larger for most experiments (overall significance $p$=$1 \times 10^{-9}$). For instance, there was a much higher increase for RAD21 ($+48\%$), CTCF ($+36\%$) or POL2 ($+29.7\%$). For TFs with few binding sites (specific TFs), there was a very strong increase, as illustrated for ATF3 ($+2323.8\%$), ETS1 ($+133.6\%$), and REST ($+794.2\%$). We explored whether evolutionary distance affects performance by using increasing number of pseudolabeled genomes (5, 10, 15, 20, and 22) sorted by phylogenetic distance (Fig. S2). Globally, we observed that for most specific TFs (ATF3, ETS1, and REST), using the five most related genomes improved AUPR, but adding more genomes (which are less related) further improved AUPR. Conversely, for other experiments, adding other genomes than the five most related genomes only slightly increased AUPR, except for ATAC-seq.

We then compared SL with SSL for DeepSea. In term of AUROC (Fig. S1), we found a significant increase using SSL ($p$=$3 \times 10^{-9}$). A large increase was found with SSL compared to SL for REST ($+23.8\%$) and P300 ($+17.5\%$). But for other data, gains were slight, around $1-2\%$. In term of AUPR (Fig. 2), as with DeepBind, improvements were larger for most experiments ($p$=$6 \times 10^{-6}$). For instance, there was a high increase for ATAC ($+42.7\%$). As for DeepBind, larger increases were found for specific TFs, e.g. ATF3 ($+148.9\%$), ETS1 ($+128.7\%$), and REST ($+1473\%$).

For both DeepBind and DeepSea, we observed a negative log-log relation between the number of positive sequences in the data and the mean AUPR increase using SLL compared to SL (DeepBind: $R$=−0.853, $p$=0.002; DeepSea: $R$=−0.713, $p$=0.021) (Fig. S3), confirming that SSL was more beneficial in the low data regime, e.g. with less than 20 000 sequences.

Large language models (LLMs), in particular transformers, are currently the most powerful neural networks for natural language processing, and were recently adapted for DNA sequences. DNABERT2 is a transformer-based LLM which was previously pretrained by self-supervised learning on genomes from 135 species, comprising 32.49 billion bases. Pretrained DNABERT2 can then be fine-tuned on a given labeled data.

Here, we used pretrained DNABERT2 to be further trained by SL or by SSL. SL consisted in fine-tuning pretrained DNABERT2 on labeled data. SSL instead consisted in further pretraining of pretrained DNABERT2 with pseudo-labeled data and then fine-tuning on labeled data (Fig. 2). Overall, there was a significant increase for AUROC (p$=0.002$) (Fig. S1), but not for AUPR (p$=0.963$) (Fig. 2). More specifically, we found that SSL strongly increased AUROCs and AUPRs for specific TFs ATF3 (AUROC: $+2.2\%$, AUPR: $+18.8\%$), ETS1 (AUROC: $+4.2\%$, AUPR: $+62.9\%$), and REST (AUROC: $+10\%$, AUPR: $+118\%$), whose data for fine-tuning are small (less than 10K peaks). For P300, we also observed increased AUROCs and AUPRs (AUROC: $+6.1\%$, AUPR: $+8.5\%$), but for other data, we found a decrease in AUROC and AUPR values. We have also compared DNABERT2 trained by SSL with the five closest species genomes (Fig. S4). We found that using 22 species genomes improved performances for specific TFs ATF3, ETS1, ANDR, and REST, as compared to only using the five closest species genomes. Such results suggest that SSL is beneficial for pretrained LLMs when fine-tuning is done on small data, which is the case for specific TFs.

### Small models trained with SSL versus large language model

In order to demonstrate the power of our new training approach, we then compared simple CNNs trained with SL, SSL or SLL-NS, with state-of-the-art large language model DNABERT2 fine-tuned without SSL for different datasets. The simple CNNs only comprised 7K parameters (CNN-7K) or 27K parameters (CNN-27K), while DNABERT2 had around 117M parameters, respectively (therefore with a magnitude of order 3–4 compared to the CNNs).

When comparing AUROCs (Table S2), we found that the simple CNN-27K trained with SSL (CNN-27K-SSL) ranked first for 4 out of 10 data, while CNN-27K trained with SSL-NS (CNN-27K-SSL-NS) also ranked first for 4 data, compared to DNABERT2 which ranked first for 2 data. For the AUPR which better accounts for class imbalance (Table 2), CNN-27K-SSL also ranked first for 4 out of 10 data, while CNN-27K-SSL-NS ranked first for 5 data. We could see that AUPR improvements were huge with CNN-27K-SSL and CNN-27K-SSL-NS, when compared to CNN-27K with SL (CNN-27K-SL), in particular for data with few peaks (small data). For instance, for ATF3, a TF that only binds to 1677 peaks, the AUPR was 0.149 for CNN-27K-SSL and 0.176 for CNN-27K-SSL-NS, which was 7 and 8 times higher than the AUPR for CNN-27K-SL (0.021), respectively. There was also an important increase for ETS1 with an AUPR of 0.139 for CNN-27K-SSL and 0.183 for CNN-27K-SSL-NS compared to 0.030 for CNN-27K-SL, and for REST with an AUPR of 0.253 for CNN-27K-SSL and 0.246 for CNN-27K-SSL-NS compared to 0.062 for CNN-27K-SL. When looking at AUPR values with scatterplots (Fig. S5), we could confirm that SSL was significantly improving CNN-7K (intercept=+0.067, $p$=$5 \times 10^{-6}$) and CNN-27K (intercept=+0.081, $p$=$3 \times 10^{-5}$), that CNN-27K-SSL significantly outperformed DNABERT2 (intercept=+0.08, $p$=0.006), and that CNN-27K-SSL-NS slightly improved CNN-27K-SSL (intercept=+0.024, $p$=0.045) and outperformed DNABERT2 (intercept=+0.103, $p$=0.001). For CNN-27K-SSL-NS, we have explored different hyper-parameter values (k: number of iterations, and SD: standard deviation of noise), and globally did not observed a strong influence of those parameters (Fig. S6).

**Table 2:** Benchmarking with AUPR curve of a simple CNN trained with SL, SSL, or SSL-NS, and comparison with large language model DNABERT2 on different datasets. AUPR: area under the precision recall curve. Each model was run three times and the average statistics were computed

AUPR							
Data	Number of peaks	Simple CNN 7K parameters supervised Learning	Simple CNN 7K parameters SSL	Simple CNN 27K parameters supervised Learning	Simple CNN 27K parameters SSL	Simple CNN 27K parameters SSL-NS	DNABERT2 117M parameters
ATF3	1677	0.008	0.124	0.021	0.149	**0.176**	0.039
ETS1	4120	0.035	0.103	0.030	0.139	**0.183**	0.052
ANDR	4638	0.002	0.0041	0.002	**0.0042**	0.003	0.002
REST	6119	0.044	0.204	0.062	**0.253**	0.246	0.015
MAX	13 605	0.077	0.104	0.087	0.1100	0.109	**0.1103**
P300	14 223	0.006	0.030	0.012	**0.043**	0.036	0.012
RAD21	34 623	0.283	0.303	0.257	**0.331**	0.324	0.172
CTCF	72 779	0.404	0.461	0.414	0.4835	**0.4844**	0.308
H3K4me3	25 641	0.128	0.156	0.154	0.165	0.170	**0.188**
POL2	35 982	0.118	0.143	0.132	0.159	**0.166**	0.131
ATAC	102 030	0.083	0.109	0.086	0.119	**0.121**	0.112
#1st place		0	0	0	4	**5**	2

Our benchmark showed that a simple CNN with only 27K parameters could outperform in certain situations or perform as well as LLM DNABERT2 which comprises millions of parameters and was trained on whole genomes. Moreover, the results revealed that our SSL strongly improved model performance for small data (i.e. specific TFs), which is expected as deep learning models comprise large numbers of parameters and therefore need large data for parameter learning. Additionally, our modified SSL-NS training further improved predictions for ATF3 and ETS1 in term of AUPR.

### Prediction of SNP effects

**Figure 3:**
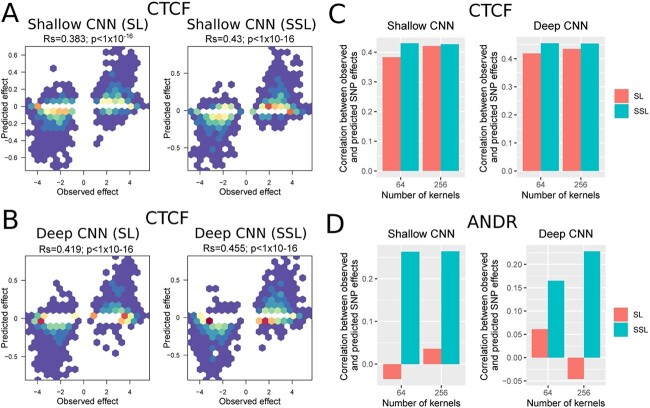
Prediction of SNP effects with SL and SSL. (A) Scatter plots of observed CTCF SNP effects versus predicted effects for a shallow CNN trained with either SL or SSL. 64 kernels were used for the convolution. An SNP effect is the observed increase (or decrease) of ChIP-seq signal of the alternative allele as compared to the reference allele. (B) Same scatter plots for a deep CNN trained with either SL or SSL. 64 kernels were used for the convolution. (C) Barplots of Spearman correlations between observed CTCF SNP effects versus predicted effects for shallow and deep CNNs, and 64 and 256 kernels. (D) Same barplots for ANDR SNP effects.

We next evaluated the ability of SSL to improve the prediction of SNP effect on molecular phenotypes such as TF binding, and compared it to SL. For this purpose, we first trained a shallow CNN with SL for CTCF peak classification, and then predicted the impact of an SNP on CTCF binding, as done in [7]. We found a good Spearman correlation between the observed effect as estimated by ChIP-seq allelic imbalance (from ADASTRA database [26]), and the predicted effect (R$s=0.383$; Fig. 3A). We then trained the same model with SSL and observed an increase of Spearman correlation (R$s=0.430$, $+12.3\%$; Fig. 3A). We repeated the same experiment with a deep CNN with dilated convolutions and residual connections and obtained an R$s=0.419$ for SL and an R$s=0.455$ for SSL ($+8.6\%$; Fig. 3B). We changed the number of convolution kernels from 64 to 256. Results showed an increase of Rs with SSL for all model predictions (Fig. 3C). We also trained CNNs to predict ANDR peaks and then predicted SNP effects. Compared to CTCF, which is a very wide-spread TF with 72 779 loci, ANDR is a more specific TF with 3415 peaks, making the model harder to train with SL as fewer data are available. Interestingly, for ANDR, we found that the predictions of SNP effects were very bad (R$s<0$) with SL, while there were positive correlations (R$s>0.25$) with SSL for shallow CNNs and (R$s>0.15$) for deep CNNs (Fig. 3D).

Results thus showed that SSL can improve the prediction of SNP effects on molecular phenotypes, such as TF binding. Moreover, we found that the gain was much higher for specific TFs for which less data were available for model training.

## Conclusion

In this article, we proposed a novel SSL based on pseudo-labeled data allowing to train models from data with multiple orders of magnitude compared to available labeled data. We further improved it incorporating principles from the Noisy Student algorithm [22] to predict the confidence in pseudo-labeled data. Such approaches alleviate the size limit of the human genomes (3.3 Gb), by leveraging other mammmalian genomes. Given that the amount of sequenced mammmalian genomes is growing exponentially due to large-scale sequencing projets (e.g. Zoonomia project [27]), such approaches represent a promising avenue for improving the performance of current deep learning models.

Training of state-of-the-art models, including DeepBind, DeepSea and DNABERT2, showed that SSL outperformed SL for DNA sequence classification in many situations, in particular when training data were small, such as for specific TFs. Moreover, SSL showed higher performances compared to SL for predicting SNP effects, especially for specific TFs. In addition, benchmarking on a diverse dataset showed that a simple CNN of 27K parameters trained with SSL performs similarly or better than LLM DNABERT2 containing 110M parameters. Globally, we found that performance gains with SSL, in particular for AUPR, was high for smaller data. Moreover, SSL combined with the Noisy Student algorithm (SSL-NS) further improved AUPRs for ATF3 and ETS1. Such results showed that the Noisy Student algorithm only improved predictions for very specific TFs, i.e. when there are very small training data. Interestingly, SSL has not improved LLM DNABERT2 much. This is likely due to the fact that DNABERT2 has already learned meaningful DNA representations with motifs, and therefore additional pseudolabeled data are not further improving the model training.

The proposed SSL approach is very flexible, as it does not require to modify a given model by adding a graph neural network layer as previously proposed in [18]. In addition, this work is related to phylogenetic data augmentation with evolutionarily related sequences from other species which was shown to improve the performance of deep learning models trained on regulatory genomic sequences [19]. Another data augmentation strategy was proposed by [20, 21] and relies on simulating the DNA sequence evolutionary process with mutations including translocations, insertions, deletions, inversions, and mutations.

SSL is not the first approach that leverages genomes without functional data. SSL with LLMs were recently trained on multiple genomes without labeled data, as a pretraining step before a fine-tuning step [11–16]. While such approach showed great performances, the training of large models (>100M parameters) on a large quantity of data (several complete genomes) requires important computational ressources, i.e. a cluster of GPUs or TPUs. SSL provides a complementary approach to pretrained LLMs by improving the accuracy of classical deep learning models (e.g. non-LLMs, for instance CNNs) in particular for specific TFs in term of AUPR. Compared to pretrained LLMs, SSL does not require to train large models with hundreds of millions of parameters. Moreover, by focusing only on homologous sequences from unlabeled genomes (i.e. only a small fraction of the genomes) and not the entire unlabeled genomes as LLMs, SSL trains models from more informative DNA sequences. Of note, the assets of SSL comes at the cost of task specificity, since the pretraining with pseudo-labeled data improves fine-tuning only for a given task. Conversely, pretrained LLMs have the main advantage to be fine-tuned on any task. Moreover, pretrained LLMs have zero-shot capabilities, i.e. for task for which the model hasn’t seen any data [28].

There are several limitations of the proposed approach. First, the pseudo-labeling assumes that the homogous sequence has the same label as the original sequence from which liftover was done. If the assumption does not hold, then SSL is expected to yield poor results, worst than those obtained with SL. Second, the use of our approach is likely limited to species which are not too evolutionary distant. For instance, it is very unlikely that including plant genomes would help to predict regulatory sequences from a mammalian genome.

**Key Points**

- Cross-species pseudo-labeling improves the training of deep learning models for regulatory sequence prediction by augmenting the data.
- Semi-supervised learning (SSL) is proposed to pretrain a model with pseudo-labeling data and then to fine-tune it on original labeled data.
- SSL shows strong performance increase for transcription factors with few binding sites (few labeled data) compared to classical supervised learning.
- A simple convolutional model (27K parameters) trained with SSL could outperform in certain situations or perform as well as large model DNABERT2 (117M parameters). In very low data regime, SSL combined with the Noisy Student algorithm (SSL-NS) further improves predictions.

## Supplementary Material

article_SSL_supp_bbae560

## Data Availability

All the code and data are available at: https://forgemia.inra.fr/raphael.mourad/deepssl.
