# Supplementary material for: Semi-supervised learning with pseudo-labeling compares favorably with large language models for regulatory sequence prediction
Source: Brief Bioinform. 2024 Nov 2;25(6):bbae560. doi: 10.1093/bib/bbae560 (PMC11531863; doi:10.1093/bib/bbae560)
Supplement: article_SSL_supp_bbae560 [file article_ssl_supp_bbae560.pdf]

Supplementary Figures

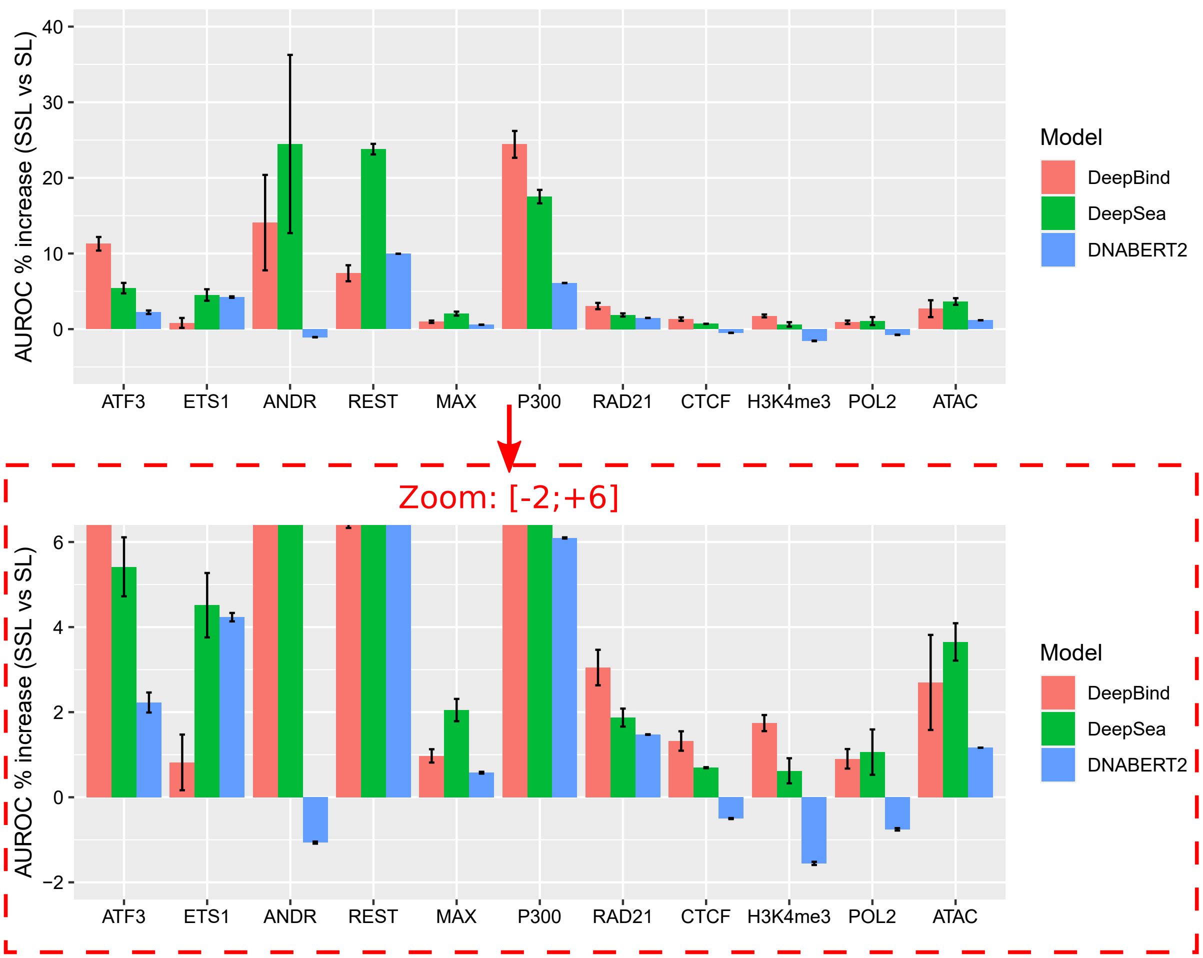

Figure S1: Percent of area under the receiver operating characteristic (AUROC) increase using semi-supervised learning (SSL) as compared to supervised learning (SL). Benchmarking was done for DeepBind, DeepSea and DNABERT2. Each model was run 3 times and the average statistics were computed.

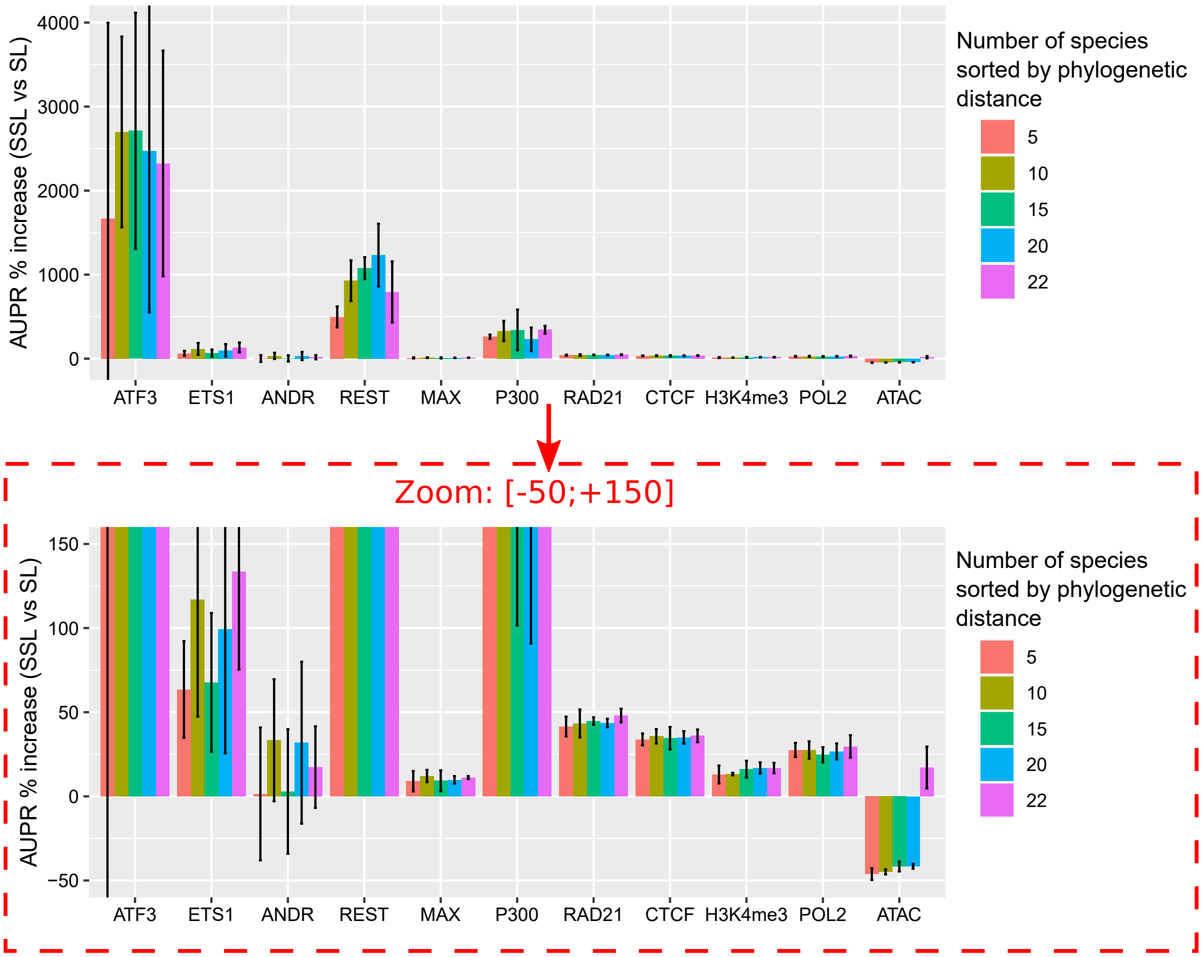

Figure S2: Percent of area under the precision recall curve (AUPR) increase using semi-supervised learning (SSL) as compared to supervised learning (SL) for DeepBind depending on the number of pseudolabeld genomes sorted by phylogenetic distance. Each model was run 3 times and the average statistics were computed.

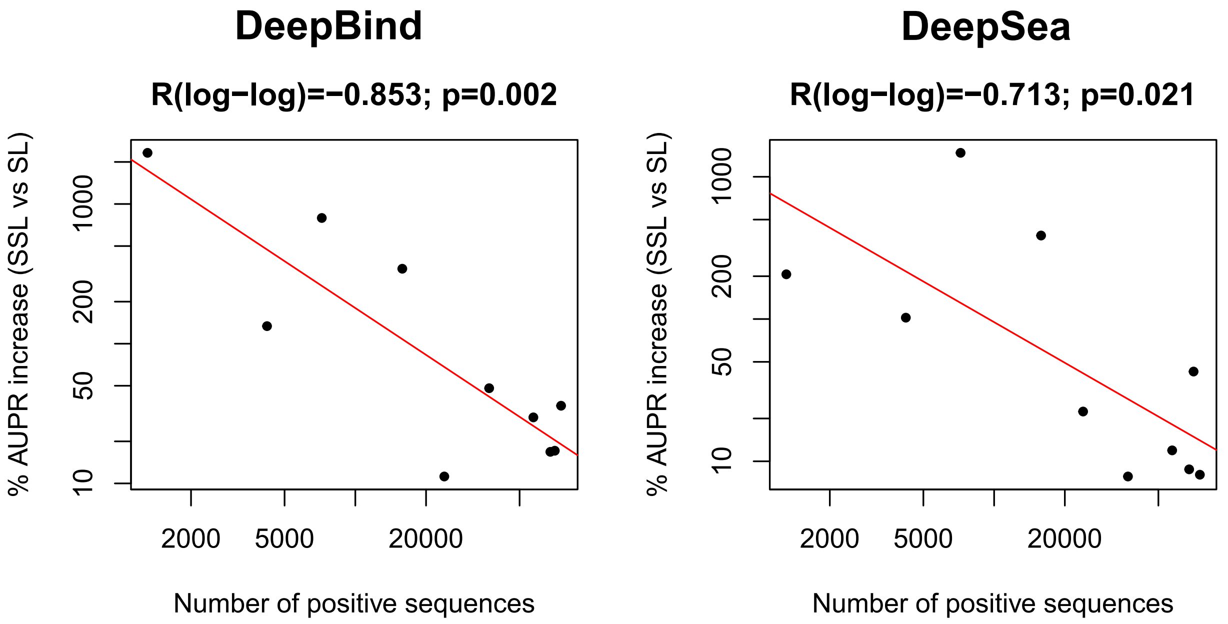

Figure S3: Mean area under the precision recall curve (AUPR) depending on the number of positive sequences for each data, for DeepBind and DeepSea.

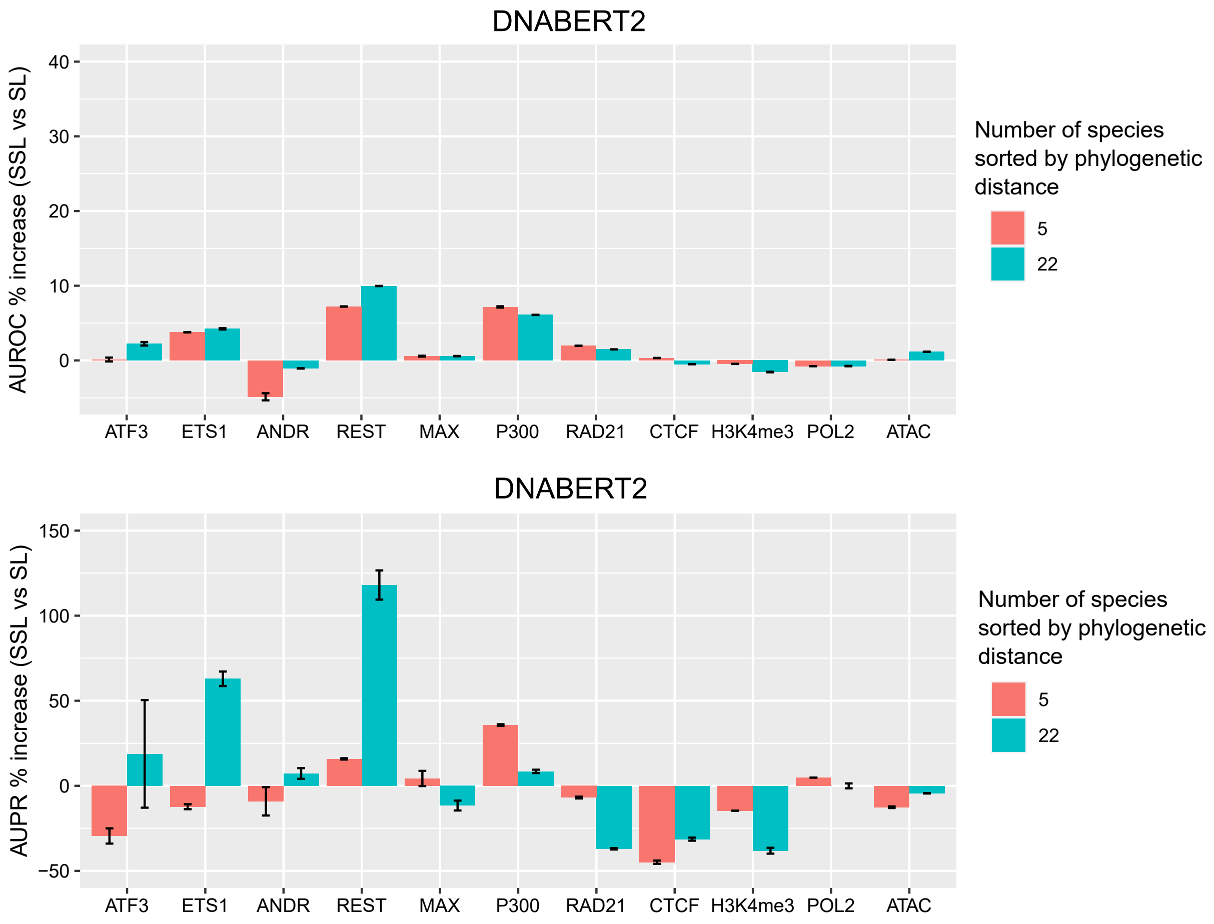

Figure S4: Percents of area under the receiver operating characteristic (AUROC) increase and area under the precision recall curve (AUPR) increase using semi-supervised learning (SSL) as compared to supervised learning (SL) for DNABERT2 depending on the number of pseudolabeld genomes sorted by phylogenetic distance (5 vs 22). Each model was run 3 times and the average statistics were computed.

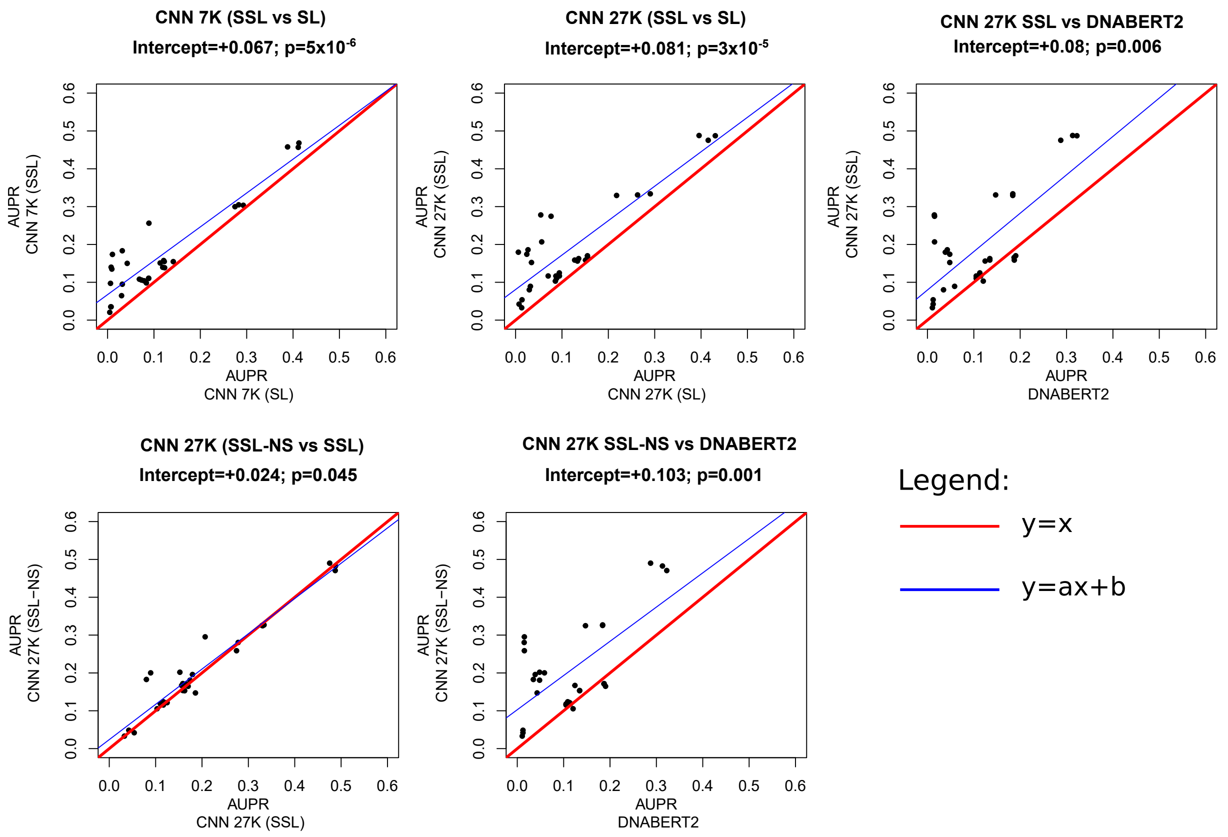

Figure S5: Scatterplots of area under the precision recall curve (AUPR) values for the following comparisons: CNN 7K SSL vs CNN 7K SL, CNN 27K SSL vs CNN 27K SL, CNN 27K SSL vs DNABERT2, CNN 27K SSL-NS vs CNN 27K SSL, CNN 27K SSL-NS vs DNABERT2.

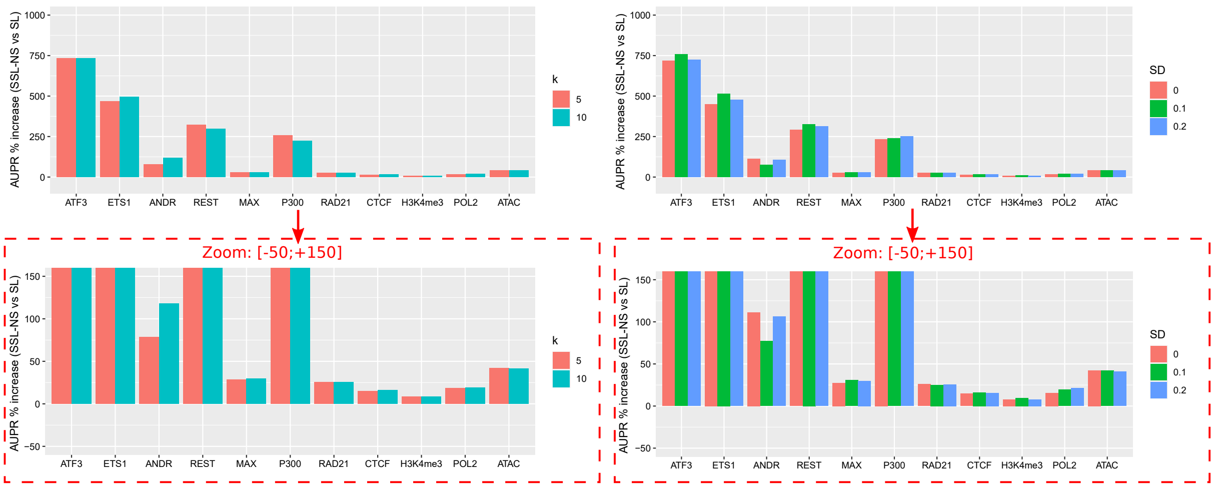

Figure S6: Percent of area under the precision recall curve (AUPR) increase using semi-supervised learning with noisy student (SSL-NS) as compared to supervised learning (SL) for a shallow CNN with 27K parameters, depending on the number k of iterations, and the standard deviation of noise (SD). Each model was run 3 times and the average statistics were computed.

Supplementary Tables

Assembly	ATF3	ETS1	ANDR	REST	MAX	P300	RAD21	CTCF	H3K4me3	POL2	ATAC
hg38	1306	4211	4638	7201	23916	15844	37113	75082	67589	57226	70600
rheMac10	1245	4045	2170	6920	23376	14948	35462	69550	65456	54851	66564
calJac3	1132	3729	1806	6497	21547	13537	32312	62203	59784	49278	59356
panTro6	1307	4091	2421	7051	23487	15412	36560	72564	66144	55653	68581
panPan2	1152	3810	2170	6856	22311	15043	36036	69263	63164	52665	65374
ponAbe3	1241	4063	1940	7014	23257	15144	36204	71008	65212	55269	67551
gorGor6	1142	3755	2024	6663	21706	14661	35038	68259	61474	50995	65297
papAnu4	1211	4030	2176	6928	23051	14841	35132	68832	64388	53714	65679
macFas5	1217	3942	2136	6834	22650	14757	34890	68362	63686	52956	64901
saiBol1	1014	3343	1644	6197	19857	13517	31910	60082	56446	46063	56595
nomLeu3	1127	3693	2189	6551	21337	14428	34115	65793	60232	50258	62600
micMur2	1034	3368	1409	5557	19013	11458	27381	51671	52853	42270	49321
otoGar3	1059	3591	1581	5947	20356	11976	28295	54181	55780	44827	51731
mm10	900	3038	1043	4688	16535	8931	20552	39691	44889	34911	38218
rn7	897	3048	1069	4614	16411	8834	20294	39372	44773	34752	38211
musFur1	952	3135	1359	5547	18349	11493	27393	50892	50850	40533	47556
oryCun2	794	2743	995	4570	14860	9309	23085	41619	40612	32169	39561
susScr11	1095	3634	1385	5805	20429	11589	27603	52724	56042	44628	50710
felCat9	1151	3734	1483	6071	21206	12248	29252	55993	57561	46148	53139
canFam3	959	3306	1359	5520	18469	11786	27916	51352	48922	40708	47874
equCab3	1173	3737	1537	6236	21425	12791	30697	58719	57902	47598	55434
bosTau9	1090	3611	1405	5764	20396	11616	26849	51766	55694	44575	50107
monDom5	663	2215	863	2785	11267	4604	9519	21299	28625	23286	22590

Table S1: Number of positive sequences and pseudo-labeled positive sequences obtained by pseudo-labeling for each of the 22 mammalian genomes and for each data.

AUROC							
Data	Number Of peaks	Simple CNN 7K parameters Supervised Learning	Simple CNN 7K parameters SSL	Simple CNN 27K parameters Supervised Learning	Simple CNN 27K parameters SSL	Simple CNN 27K parameters SSL-NS	DNABERT2 117M parameters
ATF3	1677	0.942	0.954	0.941	0.962	**0.962**	0.932
ETS1	4120	0.957	0.969	0.966	**0.974**	0.969	0.933
ANDR	4638	0.693	**0.742**	0.676	0.739	0.725	0.700
REST	6119	0.880	0.906	0.890	0.909	**0.911**	0.837
MAX	13605	0.928	0.932	0.931	**0.936**	0.929	0.936
P300	14223	0.759	0.881	0.814	**0.904**	0.901	0.834
RAD21	34623	0.883	0.897	0.881	**0.905**	0.892	0.884
CTCF	72779	0.939	0.944	0.940	0.945	**0.948**	0.932
H3K4me3	25641	0.902	0.915	0.914	0.917	0.920	**0.930**
POL2	35982	0.908	0.912	0.912	0.916	0.919	**0.920**
ATAC	102030	0.868	0.893	0.873	0.902	**0.903**	0.877
#1st place		0	1	0	**4**	**4**	2

Table S2: Benchmarking with area under the receiver operating characteristics curve (AUROC) of a simple convolutional neural network (CNN) trained with SL, SSL or SSL-NS, and comparison with large language model DNABERT2 on different datasets. AUROC: area under the receiver operating characteristics curve. Each model was run 3 times and the average statistics were computed.
